# Open-source software for automated rodent behavioral analysis

**DOI:** 10.3389/fnins.2023.1149027

**Published:** 2023-04-17

**Authors:** Sena Isik, Gunes Unal

**Affiliations:** Behavioral Neuroscience Laboratory, Department of Psychology, Boğaziçi University, Istanbul, Türkiye

**Keywords:** behavioral analysis, open-source, object tracking, animal tracking, artificial intelligence

## Abstract

Rodent behavioral analysis is a major specialization in experimental psychology and behavioral neuroscience. Rodents display a wide range of species-specific behaviors, not only in their natural habitats but also under behavioral testing in controlled laboratory conditions. Detecting and categorizing these different kinds of behavior in a consistent way is a challenging task. Observing and analyzing rodent behaviors manually limits the reproducibility and replicability of the analyses due to potentially low inter-rater reliability. The advancement and accessibility of object tracking and pose estimation technologies led to several open-source artificial intelligence (AI) tools that utilize various algorithms for rodent behavioral analysis. These software provide high consistency compared to manual methods, and offer more flexibility than commercial systems by allowing custom-purpose modifications for specific research needs. Open-source software reviewed in this paper offer automated or semi-automated methods for detecting and categorizing rodent behaviors by using hand-coded heuristics, machine learning, or neural networks. The underlying algorithms show key differences in their internal dynamics, interfaces, user-friendliness, and the variety of their outputs. This work reviews the algorithms, capability, functionality, features and software properties of open-source behavioral analysis tools, and discusses how this emergent technology facilitates behavioral quantification in rodent research.

## Introduction

Animals exhibit a wide range of behaviors in their natural habitats (Bateson, 1990). Methodological observation and categorization of animal behavior dates back to Aristotle (384–322 BC) and Erasistratus (304–250 BC), who experimented on living animals under captivity (Hajar, 2011). These observations were historically made by manual methods, relying on the human eye. However, animal behavior is so diverse that it may not be reliably recorded and categorized even under the most controlled conditions. Low levels of inter-rater reliability between different observers limit the reproducibility and replicability of the findings (Kafkafi et al., 2018). This became a major technical challenge in modern experimental psychology and behavioral neuroscience. Commercial automated behavioral analysis tools emerged in this context, aiming to produce reliable behavior categorization in rodent research (Noldus et al., 2001). This was followed by open-source software (refer to Table 1 for a dictionary definition), which offers additional flexibility in the analyses (see Kabra et al., 2013). Here, we review open-source behavioral analysis software that enable pose estimation and behavior detection/categorization in addition to animal tracking [see Panadeiro et al. (2021) for a review of animal tracking software]. We focus on the algorithms, general capability, functionality, features, and software properties of these open-source tools with their contributions and limitations. We present a glossary of common terminology (Table 1) and a list of open-access and open-source software with their identifying features (Table 2).

**TABLE 1:** Glossary of computational terms.

Term	Definition
Open-source	“Software that is free to use and can be studied or improved by anyone because it is based on a code that anyone can use” (Cambridge Dictionary, 2023)
Open-access	“Available for everyone to use” (Cambridge Dictionary, 2023)
Algorithm	“A set of rules used to calculate an answer to a mathematical problem” (Cambridge Dictionary, 2023)
Artificial intelligence (AI)	“A research field concerned with understanding and building intelligent entities—machines that can compute how to act effectively and safely in a wide variety of novel situations” (Russell and Norvig, 2003, p. 19)
Machine learning (ML)	“Machine learning is a subfield of AI that studies the ability to improve performance based on experience” (Russell and Norvig, 2003, p. 19)
Deep learning (DL)	“Machine learning using multiple layers of simple, adjustable computing elements (e.g., artificial neurons)” (Russell and Norvig, 2003, p. 44)
Precision	“A performance measure that indicates the ratio of model predictions of a class which actually belong to the predicted class” (Géron, 2019, p. 139)
Graphical user interface (GUI)	“A way of arranging information on a computer screen that is easy to understand and use because it uses icons (pictures), menus, and a mouse rather than only text” (Cambridge Dictionary, 2023)
Source code	“The set of computer instructions that have been written in order to create a program or piece of software” (Cambridge Dictionary, 2023)
Feature extraction	“Obtaining useful information from raw data by applying a series of computations” (Russell and Norvig, 2003, p. 988)
Key-point	“Points of a shape that are prominent according to a particular definition of interestingness or saliency” (Tombari et al., 2013, p. 198)
Clustering	“An unsupervised learning technique which aims to group similar data instances together into clusters” (Géron, 2019, p. 307)
Active learning	“A learning strategy where human experts interact with the learning algorithm, providing labels for specific instances when the algorithm requests them” (Géron, 2019, p. 332)
Supervised learning	“Learning a function that maps from input to output by observing input-output pairs” (Russell and Norvig, 2003, p. 671)
Unsupervised learning	“Learning patterns in the input without any explicit feedback” (Russell and Norvig, 2003, p. 671)
Dimensionality reduction	“Reducing the number of features in the data in order to improve the performance of machine learning models, or to visualize the data” (Géron, 2019, p. 279)
Neuronal networks	“A very simplified model of our neuronal circuitry, composed of a stack of layers of artificial neurons” (Géron, 2019, p. 5)
Accuracy	“A performance measure that indicates the ratio of correctly classified data instances.” (Géron, 2019, p. 22)
Heuristics	“Experience-based techniques for problem-solving, learning, and discovery. Heuristic solutions are not guaranteed to be optimal, but heuristic methods are used to speed up the process of finding satisfactory solutions where optimal solutions are impractical” (Martí et al., 2018, p. V)
Modular programming	“A design technique that divides a complex system into several parts, where each part performs a single function, and can be developed or tested independently” (Yau and Tsai, 1986, p. 714)

**TABLE 2:** Open-access and open-source behavioral analysis software.

Software	Article	Behavioral test/use^#^	Algorithm: H^a^, ML^b^, DL^c^	General capability	Functionality and features				Software properties		
					**Input modality**	**Validation or optimization**	**Background subtraction**	**Real-time**	**Modular architecture**	**Operating systems**	**Packages**
AlphaTracker	Chen et al., 2020	Social interaction	DL	Tracking, pose estimation, behavior	RGB	Validation	Yes	Yes	Yes	Windows^+^, MacOS^+^, Linux	Python Package
AnimalTracker	Gulyás et al., 2016	Not specified	H	Tracking	Grayscale	None	Yes	Yes	Yes	Windows	GUI, JAVA
Behavior Atlas	Huang et al., 2021	OFT, rearing, grooming	ML	Behavior	RGB	Validation	No	No	Yes	Windows^+^, MacOS^+^, Linux^+^	MATLAB App
BehaviorDEPOT	Gabriel et al., 2022	OFT, EPM, NORT, fear conditioning	H	Behavior	Pose and tracking data	Validation	No	No	Yes	Windows, MacOS	GUI, MATLAB App
B-SOiD	Hsu and Yttri, 2021	Locomotion, itching, rearing, grooming	ML	Behavior	Pose and tracking data	Validation	No	Yes	Yes	Windows, MacOS, Linux	GUI, Python Package
CaT-z	Gerós et al., 2020	OFT, EPM	ML	Tracking, behavior	RGB-D	Validation	Yes	No	Yes	Windows	GUI
DBscorer	Nandi et al., 2021	FST, TST	H	Behavior	Grayscale	Validation	Yes	No	No	Windows	GUI, MATLAB App
DeepAction	Harris et al., 2022	Homecage monitoring	DL	Behavior	RGB	Validation	No	No	Yes	Windows^+^, MacOS^+^, Linux^+^	GUI, MATLAB App
DeepCaT-z	Gerós et al., 2022	Locomotion, rearing, grooming	DL	Tracking, behavior	RGB-D	Validation	Yes	Yes	Yes	Windows, MacOS	GUI
DeepEthogram	Bohnslav et al., 2021	OFT, EPM, FST, social interaction	DL	Behavior	RGB	Both	No	No	Yes	Windows, MacOS, Linux	GUI, Python Package
DeepLabCut	Mathis et al., 2018	OFT, EPM, MWM	DL	Tracking, pose estimation	RGB	Both	Yes	Yes	Yes	Windows, MacOS, Linux	GUI, Python Package
ezTrack	Pennington et al., 2019	OFT, EPM, FST, NORT, fear conditioning	H	Tracking, behavior	Grayscale	Validation	Yes	Yes	Yes	Windows, MacOS, Linux	Python Package
JAABA	Kabra et al., 2013	Locomotion	ML	behavior	Tracking data	Validation	No	No	No	Windows, MacOS, Linux	GUI, MATLAB App
LabGym	Hu et al., 2022	Test agnostic	DL	Tracking, behavior	RGB	Validation	Yes	Yes	Yes	Windows^+^, MacOS^+^, Linux^+^	GUI, Python Package
LiveMouse Tracker*	de Chaumont et al., 2019	OFT, rearing, grooming, head movements	ML	Tracking, pose estimation, behavior	RGB	Validation	Yes	Yes	No	Windows	GUI
MARS	Segalin et al., 2021	Social interaction	ML, DL	Tracking, pose estimation, behavior	RGB	Validation	No	No	Yes	Windows, MacOS, Linux	GUI, Python Package
Motr	Ohayon et al., 2013	Not specified	ML	Tracking	Grayscale	Validation	Yes	No	No	Windows, Linux	GUI, MATLAB App
OpenLabCluster	Li et al., 2022	Test agnostic	ML, DL	Behavior	Pose and tracking data	Validation	No	No	No	Windows, MacOS, Linux	GUI, Python Package
SimBA	Nilsson et al., 2020	Social interaction	ML, DL	Tracking, pose estimation, behavior	RGB	Both	No	No	Yes	Windows, MacOS, Linux	GUI, Python Package
SIPEC	Marks et al., 2022	OFT	DL	Tracking, pose estimation, behavior	RGB	Validation	Yes	No	Yes	Windows^+^, MacOS^+^, Linux	GUI, Python Package
ToxTrac*	Rodriquez et al., 2017	Not specified	H	Tracking	Grayscale	None	Yes	Yes	No	Windows	GUI
TREBA	Sun et al., 2021	Not specified	H, DL	Behavior	RGB	Validation	Yes	No	No	Windows^+^, MacOS^+^, Linux^+^	Python Package
VAME	Luxem et al., 2022a	Locomotion, OFT, rearing, grooming	ML, DL	Behavior	Pose and tracking data	Validation	No	No	No	Windows, MacOS, Linux	Python Package
VSAMBR	Jhuang et al., 2010	Homecage monitoring	ML	Tracking, behavior	Grayscale	Validation	Yes	No	Yes	Windows^+^, MacOS^+^, Linux	GUI, MATLAB App

^#^Please note that specified behavioral tests are those mentioned in the original articles. Listed software may analyze other behaviors or tests that are not specified here.

^a^Hand-coded heuristics.

^b^Machine learning (non-neural network).

^c^Deep learning (neural network).

*These software are open-access, but not open-source.

^+^Software may work in these operating systems, but this is not specified in the original article.

OFT, open field test; EPM, elevated plus maze; FST, forced swim test; MWM, Morris water maze; NORT, novel object recognition test.

Designating timescales for specific behaviors, that is deciding when a particular action or movement begins and when it ends, is a key component of behavioral analysis. Manual identification of animal behavior is especially vulnerable to time-scaling differences due to potentially low levels of inter-rater reliability. This issue became a major pushing force for automated behavioral analysis software that emerged in the 1990’s. The advancement and accessibility of object tracking and pose estimation technologies gave rise to AI-based (Table 1) behavioral analysis programs that offer substantially more precise time-scaling compared to manual analysis (Luxem et al., 2022b). These open-source software utilize a variety of algorithms (Table 1) produced from non-neural network machine learning (Table 1), and deep learning (Table 1) that utilize neural networks (Table 1). These algorithms have basic differences in their internal dynamics, generalization capabilities on different settings and species, and computational costs. They use different types of graphical user interface (GUI; Table 1) and vary in user-friendliness and the diversity of outputs. Researchers either select and use the original software or modify them for their particular experimental needs.

Automated behavioral analysis often starts with tracking target objects on a two- or three-dimensional field (Jiang et al., 2022). This is known as object tracking, the ability of a software to follow the movements of an object of interest. This can be the whole animal, its extremities or other body parts such as the vibrissae (whiskers). Tracking software record the trajectories of these moving “objects” and use them to designate and categorize specific behaviors. These behaviors range from simple motor actions such as grooming and rearing to well-defined behavioral patterns such as thigmotaxis in an open field. Video-based monitoring of the posture and locomotion of the animal is employed in several behavioral mazes and tasks. The use of this technology is not restricted to rodent experiments. It has been tested with primates (Marks et al., 2022), broiler chickens (Fang et al., 2021), zebra fish (Li et al., 2022), and fruit flies (Mathis et al., 2018) as well as human eye movements (Dalmaijer et al., 2014).

The first proprietary software (Noldus et al., 2001) to utilize object tracking and monitoring technologies for rodent behavioral analysis was released in 2001 with a comprehensive behavioral repertoire that works with several paradigms. In addition to dry mazes in which the background is static, consistent results were produced in water-based paradigms such as the Morris water maze (Morris, 1981). It should be noted that the versatile protocols and user-friendly nature of this licensed software have proved success beyond the controlled laboratory settings. It has been used to track and analyze human locomotion and behaviors, such as studies on children with autism spectrum disorder (Sabatos-DeVito et al., 2019). Proprietary software, however, do not allow users to modify their algorithms or interface according to their specific needs.

Progress in the field of AI and video monitoring technology gave rise to open-access and open-source systems (Table 1) that can be downloaded, installed and modified *via* GitHub or other websites provided by the authors. The main difference between open-access repositories and open-source software is that the former does not necessarily share source codes (Table 1). The versatile nature of rodent behavioral testing often requires researchers to try new analysis on behavioral data, which do require full control over used algorithms. User-specific modifications may be needed in several different features of the analysis software, including feature extraction (Table 1), behavior classification and image pre-processing techniques. Only open-source software that provide public access can fully meet this need. These non-commercial and open-source alternatives possess the capacity to outperform commercial systems by themselves or when supported with additional machine learning classifiers (Sturman et al., 2020).

Below section reviews different groups of algorithms utilized by behavioral analysis software. This is followed by the General Capability section covering the three main components of rodent behavioral analysis: tracking, pose estimation, and behavior detection and categorization. We then go over specific features and functionality that differentiate currently available open-source software, and discuss their contribution to the main analysis tasks. In the final section, we examine software properties that relate to the design and usability of the reviewed tools.

## Algorithms

Behavioral analysis tools reviewed in this work vary in their algorithms that underly their computer vision tasks (Table 2). These algorithms can be studied in three major categories: hand-coded heuristics, non-neural network machine learning, and deep learning (neural networks). They have different hardware requirements, computational costs, and generalization capabilities, in addition to differences in data size and manual labor requirements.

### Hand-coded heuristics

Image processing combined with heuristics is the oldest approach, for which the experts extract relevant tracking and pose data (e.g., animal position, orientation, etc.) from video images by applying a series of transformations and computations like masking, thresholding, and frame differencing. They then analyze the extracted information with a set of hand-coded rule-based heuristics. These heuristics and image processing steps are defined by relatively limited experimental input, as it is not feasible to define rules that cover all possible experimental settings and scenarios. This decreases the generalizability of outputs to novel experimental data. DBscorer (Nandi et al., 2021), for instance, only offers mobility and immobility statistics by using image processing and heuristics. Although these heuristics can be extended and optimized over time by experts, as offered by BehaviorDEPOT (Gabriel et al., 2022), covering more experimental settings and behaviors is a time-consuming endeavor.

Designing new heuristics require good level of programming knowledge as well as an understanding of behavioral data of interest. BehaviorDEPOT (Gabriel et al., 2022) offers extensive support through its modules to make the heuristics design and optimization process easier. Other software like AnimalTracker (Gulyás et al., 2016), ToxTrac (Rodriquez et al., 2017), and ezTrack (Pennington et al., 2019) only offer a set of parameters that can be modified with already available data. As compared to data-hungry methods, a key advantage of heuristics-based approaches is their need for minimal, if any, annotated data. This advantage is clearly visible in BehaviorDEPOT (Gabriel et al., 2022), which shows significantly higher F1 scores compared to the machine learning-based JAABA (Kabra et al., 2013) while using less data.

Image processing and heuristics-based tools also require less computational resources. This allows AnimalTracker (Gulyás et al., 2016), ToxTrac (Rodriquez et al., 2017), and ezTrack (Pennington et al., 2019) to be adapted for real-time (online) behavioral analysis. ToxTrac (Rodriquez et al., 2017) shows outstanding performance in processing speed while having on par accuracy scores with other tracking tools. DBscorer (Nandi et al., 2021) and BehaviorDEPOT (Gabriel et al., 2022), however, are not ideal for real-time analysis, as they require tracking and pose data from DeepLabCut (Mathis et al., 2018), a deep learning-based tracking tool. These properties make heuristics-based tools suitable for researchers who work with relatively stable experimental conditions and lack sufficient annotated data or computational power required for machine learning or and deep learning models.

### Machine learning (Non-neural network)

Low levels of generalizability and other limitations of rule-based systems gave rise to a new approach that utilizes probability and statistics to create models that are able to learn and generalize from data (Russell and Norvig, 2003). Certain analysis software such as CaT-z, Motr, VSAMBR, LiveMouseTracker use a combination of image processing and classical machine learning methods. They use image processing methods to extract features and feed them into the machine learning models. One of the earliest examples of this combined use is VSAMBR (Jhuang et al., 2010), which, by integrating machine learning to its image processing capabilities, outperforms human annotators in behavioral analysis. Consisting of feature computation and classification modules, this tool extracts the motion and position features of the animal by analyzing differences in each frame and uses the extracted features to train a Support Vector Machine-Hidden Markov Model (SVM-HMM). Feature extraction with image processing, followed by a machine learning model works well for behavior classification when the annotated data is scarce. More recent tools such as Live Mouse Tracker (de Chaumont et al., 2019) and CaT-z (Gerós et al., 2020) additionally make use of RFID sensors and infrared/depth RGBD cameras to extend the features that machine learning models use to classify animal behaviors. This adds depth information to processing and substantially improves performance. Software like JAABA (Kabra et al., 2013) use machine learning-based tracking tools like Motr (Ohayon et al., 2013) to train their models. However, as discussed in the following sections, deep learning-based tracking models often yield more reliable results. Even rule-based models can be effective by using deep learning tools for tracking as observed in BehaviorDEPOT (Gabriel et al., 2022).

Compared to heuristics-based tools, machine learning systems do not require additional rule design and manual labor when a new variable, like a behavior category, is introduced to the experiments. This enables researchers to analyze a wide range of behaviors with less time and effort. This constitutes the main advantage of machine learning-based tools over rule-based methods, which require manual parameter optimization and calibration for each experimental setting. On the other hand, machine learning algorithms may perform worse compared to deep learning-based tools when there is sufficient amount of annotated data.

### Deep learning (Neural network)

Deep learning, or deep structured learning, is a type of machine learning that utilizes artificial neural networks (Table 1) with multiple layers of processing. These algorithms typically provide better key-point extraction and pose estimation. However, unlike classical machine learning and heuristic-based methods, deep learning models generally require training with large datasets to approximate the desired feature space from the given input and output pairs when they are trained from scratch (Goodfellow et al., 2016). Their behavioral analysis performance drops when the neural networks are fed solely with image data without prior feature extraction. Therefore, researchers often use a pre-trained deep learning-based model to extract trajectory and pose information, and then feed this information into other models that specialize in behavioral detection and categorization. Deep learning-based software VAME (Luxem et al., 2022a) and OpenLabCluster tackle these problems by utilizing previously extracted features (Li et al., 2022). These systems have proven to work well even when the annotated dataset is small. LabGym uses another technique to solve this common problem of neural networks (Hu et al., 2022). It removes the background from the videos, which allows models to learn features better from relevant signals by eliminating noise, and then extracts animation and positional changes of the animal. In addition to using pre-trained model weights, DeepEthogram (Bohnslav et al., 2021) and DeepCaT-z (Gerós et al., 2022), respectively, incorporate optic flow and depth information into the video frames to overcome the need for large datasets. TREBA (Sun et al., 2021) proposes a unique approach that utilizes both expert knowledge and neural networks. This method incorporates the outputs of MARS (Segalin et al., 2021) and SimBA (Nilsson et al., 2020) together with custom heuristics written by domain experts. Computed attributes are then used to train a neural network in a semi-supervised fashion, resulting in a 10-fold reduction of annotation requirements.

Recent progress in deep learning allowed software such as SimBA (Nilsson et al., 2020), B-SOiD (Hsu and Yttri, 2021), MARS (Segalin et al., 2021), and Behavior Atlas (Huang et al., 2021) to use pre-trained pose estimation and motion tracking tools for feature extraction, instead of depending on image processing or machine learning models. This enables them to combine the generalization capabilities of deep learning algorithms with classical machine learning methods that yield more robust analyses of animal behavior. In addition, Behavior Atlas (Huang et al., 2021) uses multiple cameras to estimate the 3D motion and posture of the animal and discriminates behaviors by using machine learning techniques like dimensionality reduction and unsupervised clustering (Table 1).

Deep learning-based methods share the advantages of machine learning algorithms over heuristics. Owing to transfer-learning, this approach provides substantially better generalization capabilities compared to machine learning. The requirement for large datasets is often overcome by using pre-trained networks and unsupervised or semi-supervised learning algorithms that minimize the need for annotated data.

## General capability

**FIGURE 1:**
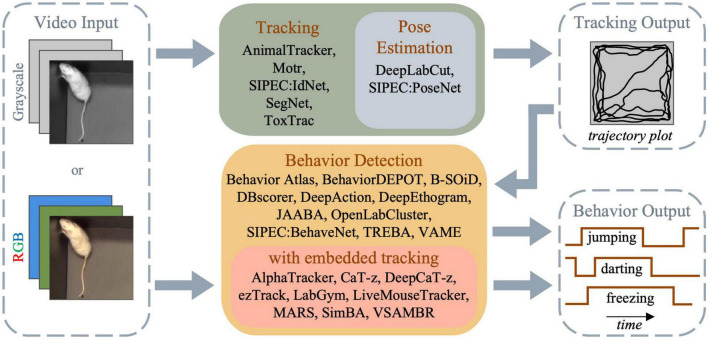
Flowchart of the general capabilities of different tracking and behavioral analysis software.

Behavioral analysis in rodent research covers tracking the locomotion (i.e., movement of the whole animal) and movement of distinct body parts to detect and categorize particular behaviors such as rearing, freezing, and thigmotaxis. Workflow of the automated analysis tools therefore consists of three main stages: object tracking, pose estimation and behavior detection/categorization. Certain software only deal with object (i.e., animal) tracking (see Panadeiro et al., 2021), while others combine tracking with pose estimation and/or behavior detection capabilities to provide automated or semi-automated rodent behavioral analysis (Figure 1).

### Tracking

**FIGURE 2:**
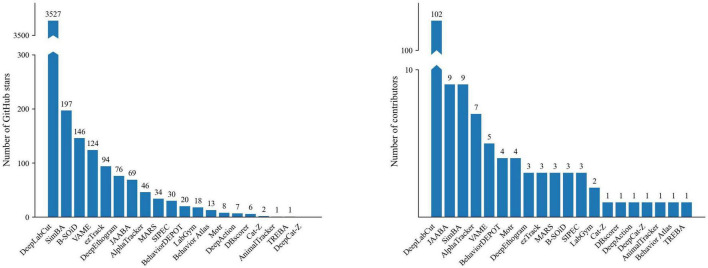
Popularity of open-source repositories as assessed by the number of GitHub stars **(left panel)** and contributors **(right panel)** as of March 2023.

Object tracking constitutes the first step for most behavioral analysis software. It includes following the moving animal and recording its position within the maze by applying different computational techniques onto the recorded image sequences. Currently, the most widely known open-source tracking tool is DeepLabCut (Mathis et al., 2018), as assessed by the number of “stars” and contributors of its GitHub repository (Figure 2). As mentioned in the previous section, it is a deep learning-based tool, which uses pre-trained neural networks and adapts them to animal tracking and pose estimation tasks by using transfer-learning. DeepLabCut is also utilized in other software like Behavior Atlas (Huang et al., 2021), BehaviorDEPOT (Gabriel et al., 2022), and SimBA (Nilsson et al., 2020). These programs focus on behavioral analysis while making use of the tracking abilities of DeepLabCut. This review focuses on open-source software that incorporate animal tracking in behavioral analysis or rely on other software for this step. However, for comparison, we also provide three examples that solely offer animal tracking without pose estimation or behavior detection/categorization: AnimalTracker and Motr are open-source tracking software, while ToxTrac is an open-access Windows tool (Table 2).

Identity preservation in multi-animal settings constitutes the most challenging sub-task of tracking. ToxTrac (Rodriquez et al., 2017), AlphaTracker (Chen et al., 2020), SIPEC:IdNet (Marks et al., 2022), MARS (Segalin et al., 2021), and LabGym (Hu et al., 2022) are capable of tracking many animals, allowing analysis of social behavior. Another software, Live Mouse Tracker (de Chaumont et al., 2019), uses RFID sensors to isolate and retain the track of different rodents. DeepLabCut (Mathis et al., 2018) recently started supporting multi-animal tracking with the help of community contributions (Lauer et al., 2022). This is another good example showing the unique strength of open-source systems: enabling custom-purpose modifications.

### Pose estimation

Pose estimation is a computer vision task that aims to encode the relative position of individual body parts of a moving animal to derive its location and orientation. This task can be carried out by deep learning models such as AlphaPose (Fang et al., 2022), a state-of-the-art whole-body pose estimation tool that can concurrently be used with many people. Deep learning-based pose estimation technology can be applied to other animals, including rodents, to facilitate behavior detection and categorization. AlphaTracker (Chen et al., 2020) utilizes the architecture of the AlphaPose to distinguish multiple unmarked animals, which appear identical. This program can therefore be utilized to study rodent social interaction, in which the body posture and head orientation signify particular types of social behavior.

As for object tracking, pose estimation algorithms of particular software can be used by other software for their particular needs. SimBA (Nilsson et al., 2020), for instance, relies on pose estimation capabilities of DeepLabCut (Mathis et al., 2018) for analyzing social interaction. Behavior Atlas (Huang et al., 2021) uses DeepLabCut to analyze videos from different viewpoints and perform 3D skeletal reconstructions from 2D pose predictions. Using multiple viewpoints and 3D pose features improves the robustness of the model against discrepancies in video recordings and obstruction of body parts.

The ResNet (He et al., 2016) architecture, a widely used pre-trained object recognition model, constitutes the backbone of DeepLabCut’s pose estimation. The last layers of ResNet are modified for a key-point detection task to reliably detect the coordinates of moving objects (i.e., animals). SIPEC:PoseNet architecture of the SIPEC (Marks et al., 2022), in contrast, utilizes a smaller network, the EfficientNet (Tan and Le, 2019), as its backbone model. With its smaller size, this model was designed to generate faster predictions compared to larger pre-trained networks like ResNet.

### Behavior detection/categorization

Detecting a particular rodent behavior and its subsequent categorization requires segmenting frames that contain specific patterns of motor actions (or lack of action/movement) across pre-defined time periods. These include species-specific responses such as freezing, darting, rearing, grooming, and thigmotaxis. As explained above, several tools like DeepLabCut (Mathis et al., 2018) employ solutions for object tracking and pose estimation, while the ultimate step, behavioral detection and categorization, is completed with a different software. Hence, the output from reliable object tracking software like Motr or more comprehensive programs that combine tracking and pose estimation are used as the input for software specialized in behavior detection (e.g., BehaviorDEPOT, JAABA, VAME, B-SOiD). This separation of functionality allows researchers to experiment with a combination of tracking, pose estimation, behavior analysis software, and find the most suitable combination for their specific needs. Certain software such as AlphaTracker (Chen et al., 2020), MARS (Segalin et al., 2021), SIPEC (Marks et al., 2022), LabGym (Hu et al., 2022), DeepCaT-z (Gerós et al., 2022), and DeepEthogram (Bohnslav et al., 2021) offer a combination of these solutions, enabling a comprehensive behavioral analysis within the same system (Table 2). This is especially useful for researchers who do not have the necessary technical skills to merge the inputs and outputs of different tools.

## Functionality and features

### Input modality

Behavioral analysis software may directly use video recordings as their input. These recordings consist of frames that can be represented in grayscale or RGB (Red, Green, Blue) color models. In a grayscale depiction, each pixel is represented by a single value that corresponds to the light intensity of that pixel, with higher values indicating a brighter pixel, and lower values indicating a darker pixel. An RGB image, in contrast, contains the hue information by representing each pixel by three values that correspond to the intensity of red, green, and blue in that pixel. Grayscale images thereby contain a single channel of information, whereas RGB images contain three channels. Grayscale images are more suitable for real-time analyses as well as offline analysis that deal only with motion capturing (e.g., color information is irrelevant). They can be acquired by monochrome cameras that possess better signal-to-noise ratio and spatial resolution in comparison to color cameras (Yasuma et al., 2010). Containing a single channel of information, grayscale images also require less computational power and time for processing. Using RGB color model in behavioral analysis may introduce additional noise to the image and decrease model performance. RGB images are useful when the color information influences the automated analysis or the observations of the researcher. Image colors can facilitate understanding how the appearance of the maze and its surroundings influence the behavior of the animal.

The performance of automated behavioral analysis programs may also vary based on the intensity and the direction of lighting in analyzed video recordings. Certain programs like ezTrack (Pennington et al., 2019) and DBscorer (Nandi et al., 2021) require a good level of contrast between the animal and the background. This can be obtained by providing sufficient illumination on the field of interest (e.g., the experimental apparatus/maze) and playing with the distance and direction of the video camera. Other software, such as CaT-z (Gerós et al., 2020), are less vulnerable to lighting conditions and deliver consistent results under non-uniform illumination. When rodents are observed through RGB cameras in a behavioral maze, the distinction between different body parts and the background may not be possible with the naked eye. Automated software substantially ameliorated this problem. Combining depth information with the RGB input of video cameras significantly improved object detection, even in relatively dark environments. With RGB-D (D stands for depth) cameras, lighting conditions ceased to be a determining factor in behavioral analysis.

Automated programs that solely focus on behavioral categorization (e.g., B-SOiD, VAME, JAABA, and BehaviorDEPOT) rely on tracking or pose estimation capabilities of other software. They do not process graphic information themselves, but utilize data extracted from the videos. When multiple behavioral analysis tools support outputs from a common tracking/pose estimation software, different analyses can be applied to the same dataset without processing the videos multiple times, saving time and energy. For example, VAME (Luxem et al., 2022a) and BehaviorDEPOT (Gabriel et al., 2022), which, respectively, rely on deep learning models and heuristics, utilize output data from DeepLabCut.

### Validation and optimization

Validation methods ensure fitness and robustness of the computational model, while optimization deals with the reliability of the model on novel data. Validation is used to verify the accuracy (Table 1) of the model by evaluating the predictions on the acquired data (Hastie et al., 2001), which informs researchers on the generalization capabilities of their model. Optimization, in contrast, refers to fine-tuning the software parameters in order to minimize model prediction errors on validation datasets (Bergstra et al., 2013). Using validation and optimization methods in animal behavioral analysis contributes to the error correction in the models and increases the reproducibility and generalizability of research findings. As shown in Table 2, the majority of open-source analysis software utilize validation methods, while some use both validation and optimization.

### Background subtraction

Background subtraction refers to the image processing technique of isolating the moving object of interest, in this case the experimental animal, from the background of the video recording. Reliable detection of the moving animal on the foreground can boost tracking, pose estimation, and behavior detection performance. Background subtraction in automated behavioral analysis software often relies on one of the two techniques: masking or object segmentation. Masking is a well-known image processing technique, used by behavioral analysis software to build robust models. It allows researchers to focus on specific regions of interest within a video frame, allowing them to isolate and categorize specific behavioral patterns. This is especially useful for behaviors or states that involve limited movement or locomotion such as freezing or immobility during the forced swim test (Unal and Canbeyli, 2019). Masking also helps to reduce the influence of extraneous variables, such as the presence of other animals or distractions in the background, on the analyzed behavior. This improves the accuracy and reliability of visual data collection and enables researchers to identify subtle behaviors that are otherwise difficult to discern. Masking is also useful in tracking the movement of individual animals within a group, as it allows researchers to isolate and analyze the behavior of a specific animal within the group composed of other moving conspecifics (Kretschmer et al., 2015). The use of masking techniques in behavioral analysis software is one way to substantially enhance the precision (Table 1) and reliability of research findings. Object segmentation, in contrast, is a relatively more complex approach, which generally utilizes deep learning models instead of simple image processing techniques of masking to separate background and foreground objects. It helps define the boundaries of the animals more accurately so that they can be separated using the masking technique.

### Real-time analysis

Usefulness of real-time analysis in behavioral neuroscience is observed in *in vivo* electrophysiological experiments that manipulate ongoing neuronal activity with closed-loop protocols. This allows researchers to combine detection of specific electrophysiological events with stimulation or inhibition (see Couto et al., 2015). Likewise, particular behaviors can be followed by neuronal manipulation with real-time detection (see Hu et al., 2022). Behavioral analysis software that can function in real-time typically utilize sensors or tracking devices attached to the animal. They provide a continuous stream of data on the locomotion of the rodent and the movement of its extremities. Real-time analyses are used to study a wide range of behaviors including social interactions, feeding patterns, and locomotor activity. Video analysis software, in contrast, can only analyze the recorded video footage of the animal. Video analysis is used for behaviors that either do not need to be detected in real-time or are difficult to detect in real-time due to their long-time span.

## Software properties

### Modularity

Modular software architecture, also known as modular programming (Table 1), is a general programming concept that involves separating program functions into independent pieces, each executing a single aspect of the executable application program (Yau and Tsai, 1986). Open-source behavioral analysis programs show key differences in modularity. BehaviorDEPOT, for instance, consists of six separate modules: the analysis module, experiment module, inter-rater module, data exploration module, optimization module, and the validation module. Such multi-functionality enables users to analyze rodent behaviors in a comprehensive way. The analysis module, for instance, uses the key-point tracking output of another program, DeepLabCut, as its input to define behaviors like escaping and novel object exploration (Gabriel et al., 2022). Another example of a modular automated analysis software is ezTrack (Pennington et al., 2019). It consists of a freeze analysis module and a location tracking module. CaT-z (Gerós et al., 2020), on the other hand, includes three different modules that take annotated recordings from RGBD sensors as input. Programs with no modular system may offer limited variety in behavioral analysis and specialize in particular paradigms. DBscorer, for instance, is only used to assess behavioral despair by recording immobility in the forced swim test and tail suspension test (Nandi et al., 2021). Furthermore, modular architectures enable researchers to use the modules separately, which decreases computational workload and saves time when analyzing large amounts of data.

### Operating systems and packages

Since users of the open-source behavioral analysis software are behavioral neuroscientists and experimental psychologists, who may lack strong programming skills, user-friendliness emerges as a key aspect of these software. In fact, one of the primary motivations for developing automated analysis software is to make behavioral analysis processes easier and faster for researchers from diverse backgrounds. It should be noted that proprietary software excels in this area, offering a costly alternative to open-source programs listed in this article (Table 2).

The most important aspect of a user-friendly software is having a stable and functional user interface. A good user interface design enables researchers to learn and use the program faster with minimal error. Another defining feature of a user-friendly software is its compatibility with different operating systems. While certain programs like DBscorer (Nandi et al., 2021) and Live Mouse Tracker (de Chaumont et al., 2019) are only optimizable in Microsoft Windows, others like ezTrack are available in all common operating systems, including Unix systems like macOS and the open-source Linux (Table 2).

Automated analysis software have certain prerequisites for reliable use of their key-point (Table 1) tracking system in pose estimation. Programs like BehaviorDEPOT (Gabriel et al., 2022), are less effected by environmental changes compared to machine learning-based software, and therefore provide a better key-point tracking performance. However, these programs also ask researchers to train the key-point tracking system (Gabriel et al., 2022), increasing user workload and analysis time. Some open-source software like ezTrack (Pennington et al., 2019) require little or no background in programming. These programs utilize simple computational notebooks or GUI designs that enable researchers to effectively use them irrespective of their level of computational literacy. Other software may offer a Python package, MATLAB App, or a combination of these (Table 2). VAME (Luxem et al., 2022a), for instance, only works *via* a custom-made Python package, while DeepEthogram (Bohnslav et al., 2021) provides a GUI in addition to a Python package.

## Conclusion

Open-source behavioral analysis software offer an affordable, or virtually free, alternative to proprietary software. Researchers still need to acquire the necessary computing power to effectively run these software, many of which require powerful, hence costly, GPUs. Avoiding software license fees, however, is a significant financial relief for many laboratories, and an important contribution to “the democratization of neuroscience” (Jackson et al., 2019).

Another major impact of the open-source software movement is its contribution to the relative standardization of behavioral analysis in rodent research. Securing a good inter-rater reliability has been a major concern in traditional rodent behavioral analysis, where at least two independent observers record and categorize behaviors. Automated analysis software eliminates the potential variability between different observers, producing substantially consistent results within each experiment. Furthermore, by using automated software, researchers/coders do not need to be blind to the experimental conditions. A complete, universal standardization of behavioral analysis is not possible, as different analysis software and their different versions may produce dissimilar results on the same behavioral experiment. Yet, accessibility of open-source software makes it possible for laboratories to easily re-analyze their results with other programs, allowing standardized comparisons between findings of different research groups. Open-source behavioral analysis software do not only facilitate behavioral/systems neuroscience research done under controlled laboratory conditions, but they also contribute to computational neuroethology, in which the environmental context is also incorporated in the model (Achacoso and Yamamoto, 1990; Datta et al., 2019).

An important move to overcome technical challenges of open-source software use would be establishing international societies and networks that aim to disseminate the know-how on designing and using these software in rodent research. The European University of Brain and Technology, NeurotechEU, is a good example in this pursuit, connecting different training opportunities and making them available to students and young scientists through its graduate school and Campus + initiative. Implementing a research vision and strategy that places programming skills and open-source software development at its core is a common trend in science (Martinez-Torres and Diaz-Fernandez, 2013). Albeit some behavioral analysis software like BehaviorDEPOT (Gabriel et al., 2022) state that a computational background is not necessary to effectively use open-source software, coding and programming already constitute a central skillset in contemporary life sciences.

Analyzing behavioral data has been a time-consuming and arduous task in rodent research for decades. Moreover, inter-rater reliability issues were a major factor in manual coding, effecting both the reproducibility and replicability of research findings. Manual methods were often insufficient to reliably capture the wide range of behaviors displayed by rodents. Various AI-based algorithms, incorporated into the open-source software reviewed in this article, became the game changer in rodent behavioral analysis. The open-source automated behavioral analysis tools offer an affordable and accessible alternative to proprietary software, while enabling custom-purpose modifications to suit unique research needs. Importantly, these tools contribute to the standardization of rodent behavioral analysis across different laboratories by eliminating the inter-rater reliability issues of manual coding. The growing use of open-source analysis software is a general trend in science and already constitutes the gold standard of analyzing rodent behaviors in contemporary neuroscience.

## Author contributions

Both authors conceptualized the work, conducted the research, wrote the manuscript, and approved the submitted version.
